# Ultrasound detection of non-atherosclerotic intima-medial abnormalities of lower limbs arteries in amateur endurance runners

**DOI:** 10.1007/s40477-024-00916-6

**Published:** 2024-07-11

**Authors:** O. Benacka, O. Jiravsky, M. Labudova, J. Benacka, E. Goncalvesova

**Affiliations:** 1 Department of Cardiology, Faculty of Medicine, Comenius University, National Cardiovascular Institute, Pod Krasnou Horkou 1, Bratislava, 83348 Slovakia; 2 Hospital AGEL Trinec Podlesi, Trinec, Czech Republic; 3 Faculty of Health Sciences and Social Work, Trnava, Slovakia; 4 Slovak Society for Ultrasound in Medicine, Piestany, Slovakia

**Keywords:** Endurance athletes, Vascular ultrasound, Non-atherosclerotic noduli, Intima-medial blurring

## Abstract

**Background:**

Structural changes in the lower limb’s arterial wall in amateur endurance runners are a rare incidental finding, represented just by several case reports.

**Aim:**

Study the incidence of non-atherosclerotic lower limb artery wall changes in defined group of amateur endurance runners and identify relationship with the training parameters and the relevant biochemical markers.

**Methods:**

Amateur male athletes engaged in endurance running for more than 5 years were enrolled. Tibial and anterior popliteal arteries on each side were examined by ultrasound with focus on non-atherosclerotic structural wall changes: intima-medial border blurring, presence and character of non-atherosclerotic noduli. Subsequently the descriptive and correlation analysis were performed.

**Results:**

The study enrolled 20 amateur male endurance runners from Black Swan Triathlon Club Slovakia. The low atherosclerotic risk was represented by normal lipid levels, BMI under 30 kg/m^2^ and non-smokers in all participants. At least one type of structural artery wall abnormality (noduli or intima-medial border blurring) was present in 19 of 20 participants (95%). The most present was the intima-medial blurring. (80% of participants). The noduli were present in 65% of study group, in almost 40% of these, they were considered as hyperechogenic. All these affections were predominantly in popliteal artery area (65%). The vast majority has bilateral affection. We find a mild correlation between these ultrasound findings and training load represented by annual kilometers and run hours. There was no association between these changes and lipid spectrum or CRP level.

**Conclusion:**

The subclinical lower limb artery changes, represented by intima-medial border blurring and non-atherosclerotic noduli were present in almost every amateur endurance runner. Despite the underlying mechanism is not understood, the increased training load seems to be one of the responsible factors.

## Introduction

In the recent years, there is increasing number of amateur athletes in general population, especially endurance runners. Therefore, a more frequent traumatic damage of lower leg soft tissue and vessels can occur. As a bystander finding on ultrasound scans of these injuries a subclinical damage of arteries has been incidentally reported. These artery wall changes are represented by unspecific noduli and intima-medial border blurring, which do not exhibit the typical signs of atherosclerotic process. Finally, the atherosclerosis is unlikely in these patients, because of younger age, healthy lifestyle, and absence of standard risk factors. Until now, these structural wall abnormalities have been reported just in several case reports. We have carried out a prospective study of amateur endurance athletes to evaluate prevalence of subclinical lower limb arterial wall changes and their relationship to training load and training characteristics.

## Methodology

This is a single-center study. We enrolled male Black Swans Triathlon Club Slovakia members, who have been engaged in endurance running for more than 5 years. The running intensity was determined by a questionnaire, focused on intensity, frequency and length of running trainings per week, number and length of competitive endurance runs and running conditions (shoes, post-load stretching, hydrotherapy or massage, surface for training runs). The anthropometric data, lipid spectrum, biochemical parameters and blood count was examined. The lower limb ultrasound examination was performed on a non-training day, with Esaote MyLabC unit (Italy) unit, with 7–13 MHz LA probe, by one investigator by predefined standard protocol. Two locations for displaying arteries on both limbs were defined: arteria tibialis anterior (ATA) and arteria poplitea (AP). We evaluated 10 cm long segment for each artery. Following ultrasound parameters were evaluated in zoom mode on each segment: minimum lumen diameter, blurring of intima-medial border, number of the noduli and echogenicity of the noduli. Blurring of intima-medial border was represented semiquantitative (0 = not present, 1 = present), number of noduli semiquantitative (0 = not present, 1 = less than five noduli, 2 = more than five noduli), echogenity of the noduli semiquantitative (1 = low echogenic, 2 = high echogenic). Ultrasound examination of carotid arteries was performed in each participant.

Data analysis was performed with IBM SPSS Statistics version 28.0.0.0. Descriptive statistics for continuous variables were presented as mean and standard deviations, categorical data were summarized as frequencies and percentages. The Shapiro–Wilk test was used to assess the normality of continuous variables. Non-parametric tests were used for variables related to running stress that deviated from a normal distribution. Pearson's correlation coefficient was used to identify linear associations between continuous variables, with significance set at p < 0.05. The Mann–Whitney U test was used to compare continuous data between two groups, and the chi-squared test was used for categorical data. 95% confidence intervals (CI) were calculated to provide a range for the true population parameters.

## Results

In our study, 20 male amateur runners were enrolled, aged from 19 to 61 years (average 44.3 years). Basic study group characteristics is described (Table 1). There was no smoker, no participant exceeded BMI more than 28 kg/m^2^, all athletes were normotensive, without positive family history of premature cardiovascular disease. All participants have normal lipid spectrum (LP(a), LDL, non-HDL, HDL / non-HDL atherogenic index). Each participant takes minimum 60 min of run training at least four times weekly (Table 2). The majority of runners train on hard surface, with air cushion trainers, just with simple muscle stretching after workout (Table 3).

**Table 1:** Characteristics of the study population

Parameter	n = 20	SD
Age [years]	44.3 (19–61)	8.6
Height [cm]	179.5 (168–190)	6.5
Weight [kg]	78.5 (64–93)	8.8
BMI [kg/m^2^]	24.3 (21.6–27.5)	1.8
HDL [mmol/l]	1.8 (1.29–2.53)	0.3
Non-HDL [mmol/l]	3.3 (2.71–4.81)	0.5
AT Index	2.0 (1.33–3.19)	0.5
Total Cholesterol	5.03 (3.86–5.55)	0.7
Lp(a) [g/l]	0.3 (0.02–1.80)	0.4

**Table 2:** List of physical activities

Parameter	n = 20	SD
Running weekly [hours]	4.1 (2–7)	1.4
Training unit length [minutes]	70.2 (60–120)	19.5
Running hours per year	246.7 (100–500)	119.8
Years of training	12.1 (5–30)	6.9
Life running hours	3175.5 (800–12000)	3082.4
Marathon sum [sum]	9.1 (0–35)	9.9
Up to 10 km runs [sum]	25.2 (4–80)	18.2

**Table 3:** Training conditions

Parameter		n	%
Running surface	Tartan and grass	6	30
	Solid surface (asphalt, concrete)	14	70
Running shoes	With air cushion	12	60
	Ordinary	8	40
Relax after running	Massage, hydrotherapy, stretching	5	25
	Only stretching	15	75

At least one type of structural artery wall abnormality (noduli, intima-medial border blurring or higher wall thickness) was present in 19 of 20 participants (95%). The most frequent was the intima-medial blurring, in 80% of participants. Noduli were present in 65% of study group, in almost 40% of them, they were considered as hyperechogenic. All these affections were predominantly in popliteal artery area (65%). The vast majority has bilateral affection. Maximum number of noduli per 10 cm segment was 8 in count. ATA minimal diameter was 2.01 mm (1.45–2.7, SD 0.3), AP minimal diameter was 5.28 mm (4.04–7.6, SD 0.8). Standard Doppler flow parameters in all participants were physiological.

**Fig. 1:**
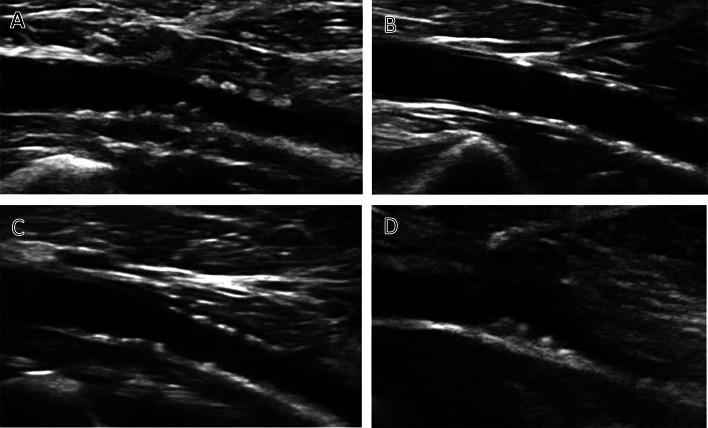
Pathological changes of the popliteal artery. A: Advanced nodular wall changes with low echogenicity. B: Flat nodules in the wall with high echogenicity. C: Numerous flat nodules with low echogenicity and narrowed lumen. D: Thick wall with nodules

The intima-medial blurring on tibial artery showed positive correlation with total running hours (*r* = 0.323) confidence index from − 0.140 to 0.670. The tibial artery minimal lumen showed negative correlation with annual running kilometers (*r* = − 0.310). No relationship was found between arterial wall changes and anthropometric or biochemistry data (e.g. complete lipid spectrum, CRP level or hepatic function). Ultrasound examination of carotid arteries showed no abnormalities in whole study group, carotic intima-medial thickness was less then 0.6 mm in all participants (Fig. 1).

## Discussion

**Fig. 2:**
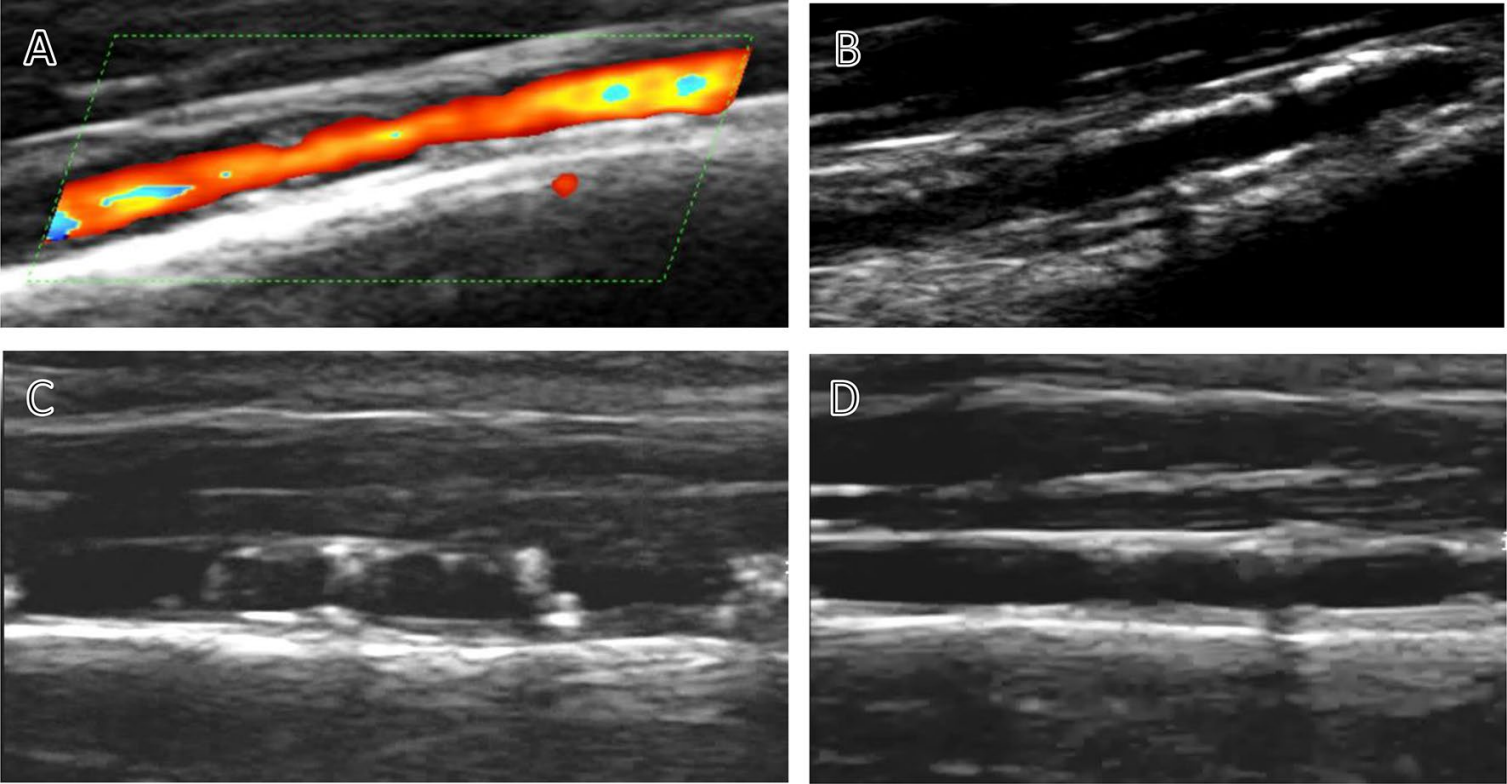
Pathological changes of the anterior tibial artery. A: Unequal changes of the artery lumen. B: Confluent echogenic wall changes. C: Hyperechogenic nodules with narrowed artery lumen. D: Advanced structural changes of thickened wall and intima-media border blurring

There are just several case reports showing similar lower limb artery changes in endurance runners. Ross et al. published for the first time a case report of three random observations of structural changes in the wall of the foreleg arteries in asymptomatic endurance runners [1]. A typical ultrasound image consisted of echogenic nodular structures embedded in the wall in the form of beads ("rosary"). The etiology, mechanism and clinical implications of these observations were not specified in his publication (Fig. 2).

Several authors focused on monitoring peripheral resistance as manifestations of functional changes in arteries during endurance exercise. Regular moderate-intensity endurance sports reduce afterload, high training increases arterial stiffness [2]. Shorter training run reduces stiffness, but a longer one worsens stiffness [3]. Other authors found no differences in arterial stiffness depending on the running intensity and duration [4] or observed after marathon decrease in the augmentation index without any arterial stiffness change [5]. All cited papers did not describe the morphology of the arterial wall with possible structural changes.

Kröger monitored atherosclerotic (AS) changes on the carotids and peripheral arteries in older marathon runners (aged 50 to 75 years), the AS changes correlated with the calcium score on the CT coronarography [6]. In our case, the probands age was significantly younger, without any AS risk factors. The morphological changes not typical for AS had not statistical relationship with lipid spectrum and HDL level in all probands is higher, primarily as an effect of regular physical activity.

There are two types of vessel calcification remodeling described: intimal and medial. The distinction between these two is possible only by histological examination. Deposition of calcium in the media is in different biochemical forms, the most common are calcifications of atherosclerotic genesis [7]. Another arterial pathological entity is fibromuscular dysplasia (predominantly by middle-aged women) affecting the medial or adventitial layers of the renal or extracranial arteries—internal carotid arteries with typical findings on CT imaging and USG, named “string-of-beads”, with higher turbulent flow velocity, involvement of the renal arteries leads to secondary hypertension [8, 9]. Other literary sources also describe the occurrence on the mesenteric arteries with M. Crohn's symptoms [10]. The wall changes observed in our study have no similarity with previously described morphological changes, all probands are normotensive. Structural artery wall changes described in our study should be caused mechanically by shocks during long-term running. Majority of our study group (only men) trained on hard surface.

Flow-limiting endo-fibrosis of the iliac arteries is a condition seen mainly in trained endurance bikers, with rises of flow velocity while no characteristic structural wall changes have been described [11, 12]. In our study, on superficial femoral artery (AFS) were wall structural changes only rarely observed, therefore the findings on the AFS were not included in the analysis.

Thijssen reported that more intense physical exercise can reduce arterial wall thickness, about arterial lumen absent data in source and little is known about the exercise intensity required to optimal benefits [13]. Our observations found other relationship: with growth of yearly running kilometers narrows the arterial lumen.

Our study has several limitations. There was no control group. We presumed no noduli and no intima-medial blurring in healthy men (non-runners) without presence of risk factors of atherosclerosis. Women were not included in the study, as there are few vigorous runners among women. Further investigation is needed to understood the pathogenesis of these findings. In the currently available literature, there are no published data about longer follow up of these wall changes and their clinical impact. It could be a challenge to follow up these probands over a longer time in order to understand the connections of mechanical overload as well as monitor morphological structural changes over time.

## Conclusion

We described the subclinical morphological non-atherosclerotic lower limbs arteries changes in group of amateur endurance runners. These changes were detected by ultrasound and were represented by intima-medial border blurring and non-atherosclerotic noduli in form of beads. These changes affected almost every participant. A possible connection to higher training load and hard run training surface can be proposed. The clinical importance is unclear. Further follow-up studies should be carried out. 
